# Automated quantification of cartilage quality for hip treatment decision support

**DOI:** 10.1007/s11548-022-02714-z

**Published:** 2022-08-17

**Authors:** Adrian C. Ruckli, Florian Schmaranzer, Malin K. Meier, Till D. Lerch, Simon D. Steppacher, Moritz Tannast, Guodong Zeng, Jürgen Burger, Klaus A. Siebenrock, Nicolas Gerber, Kate Gerber

**Affiliations:** 1 sitem Center for Translational Medicine and Biomedical Entrepreneurship, Personalised Medicine, University of Bern, Bern, Switzerland; 2 Department of Diagnostic-, Interventional- and Pediatric Radiology, Inselspital, University Hospital of Bern, Bern, Switzerland; 3 Department of Orthopaedic Surgery and Traumatology, Inselspital, University Hospital of Bern, Bern, Switzerland; 4 Department of Orthopaedic Surgery and Traumatology, Fribourg Cantonal Hospital, University of Fribourg, Fribourg, Switzerland

**Keywords:** Hip cartilage, Deep learning, Morphological, Biochemical, Automatic, Quality metric

## Abstract

****Purpose**:**

Preservation surgery can halt the progress of joint degradation, preserving the life of the hip; however, outcome depends on the existing cartilage quality. Biochemical analysis of the hip cartilage utilizing MRI sequences such as delayed gadolinium-enhanced MRI of cartilage (dGEMRIC), in addition to morphological analysis, can be used to detect early signs of cartilage degradation. However, a complete, accurate 3D analysis of the cartilage regions and layers is currently not possible due to a lack of diagnostic tools.

****Methods**:**

A system for the efficient automatic parametrization of the 3D hip cartilage was developed. 2D U-nets were trained on manually annotated dual-flip angle (DFA) dGEMRIC for femoral head localization and cartilage segmentation. A fully automated cartilage sectioning pipeline for analysis of central and peripheral regions, femoral-acetabular layers, and a variable number of section slices, was developed along with functionality for the automatic calculation of dGEMRIC index, thickness, surface area, and volume.

****Results**:**

The trained networks locate the femoral head and segment the cartilage with a Dice similarity coefficient of 88 ± 3 and 83 ± 4% on DFA and magnetization-prepared 2 rapid gradient-echo (MP2RAGE) dGEMRIC, respectively. A completely automatic cartilage analysis was performed in 18s, and no significant difference for average dGEMRIC index, volume, surface area, and thickness calculated on manual and automatic segmentation was observed.

****Conclusion**:**

An application for the 3D analysis of hip cartilage was developed for the automated detection of subtle morphological and biochemical signs of cartilage degradation in prognostic studies and clinical diagnosis. The segmentation network achieved a 4-time increase in processing speed without loss of segmentation accuracy on both normal and deformed anatomy, enabling accurate parametrization. Retraining of the networks with the promising MP2RAGE protocol would enable analysis without the need for B1 inhomogeneity correction in the future.

## Introduction

Hip cartilage quality is an important diagnostic factor in the treatment planning of hip deformities such as femoroacetabular impingement (FAI) [1, 2] and hip dysplasia [3, 4]. Left untreated, such conditions can lead to premature osteoarthritis (OA) [5, 6], requiring total joint replacement. Studies have shown that early surgical correction and chondro-labral repair can restore hip function, reduce pain, and preserve the native joint in the long term [1, 3, 7–9].

Success of preservation surgery is, however, dependent on a number of factors, including the severity of the existing cartilage damage. Numerous studies have revealed good outcomes following early surgical intervention in patients with pre-existing mild OA, and poor outcome results in patients with advanced degeneration [3, 9]. Differentiating mild OA from more advanced OA in order to identify patients likely to benefit from preservation surgery is, thus, important. Detecting cartilage damage and degeneration at an early stage in order to maximize the patient’s benefit, particularly in young patient populations, is also critical.

In clinical routine, cartilage degeneration is most commonly assessed manually as a measure of joint space on standard radiographs. However, this indirect estimation of the decrease in cartilage thickness reportedly fails to detect early degenerative changes [2]. The accuracy of radiographic measurements is also affected by factors relating to 2D projection, variations in image acquisition technique, and patient positioning [10, 11].

More recently, biochemical magnetic resonance imaging (MRI) sequences, such as the delayed gadolinium-enhanced MRI of cartilage (dGEMRIC) technique [7, 12], have proved beneficial in identifying more subtle early changes in cartilage quality [13, 14]. The protocol provides indirect quantification of glycosaminoglycan depletion, one of the earliest degenerative changes [5], and early studies have shown prognostic value for premature joint failure prediction following joint preserving surgery [5]. Cartilage degeneration in cases of malformation is not, however, spatially uniform [15], and studies have identified the importance of regional analysis [16–18]. For example, Kim *et al.* concluded that the dGEMRIC index of the anterior joint may better predict premature joint failure [8]. Acetabular cartilage degeneration also typically precedes femoral cartilage damage in FAI and hip dysplasia [19, 20]. Thus, a separate analysis of acetabular and femoral cartilage, in addition to regional analysis, would theoretically provide a better estimation of actual damage to the joint early in the degeneration process. Cartilage layers are generally not visible on 3D MRI data making them difficult to differentiate [14], and analysis of a small number of reformatted 2D images provides a limited assessment of regional differences.

In addition to biochemical analysis, morphological factors have also been associated with the success of preservation surgery. For example, the acetabular lunate surface size is an important consideration for treatment planning [21], with smaller surface areas associated with failure [22].

To enable more detailed 3D analysis of the hip cartilage, automatic segmentation has been previously proposed [23–26]. We recently demonstrated the feasibility of a deep learning-based approach for 3D analysis of dGEMRIC images of patients with FAI and dysplasia deformities [14].

This work describes a complete automated system for 3D cartilage analysis of the hip including: automatic cartilage segmentation and modelling; separation of the cartilage into analysis regions; and a fully automatic biochemical and morphological assessment of each cartilage section. Such a tool aims to provide patient-specific diagnosis and decision-making support and would facilitate standardized longitudinal analysis of cartilage composition in large prospective trials that aim to monitor the effect of hip joint preserving surgery and the natural course of osteoarthritis.

## Methods

**Fig. 1:**
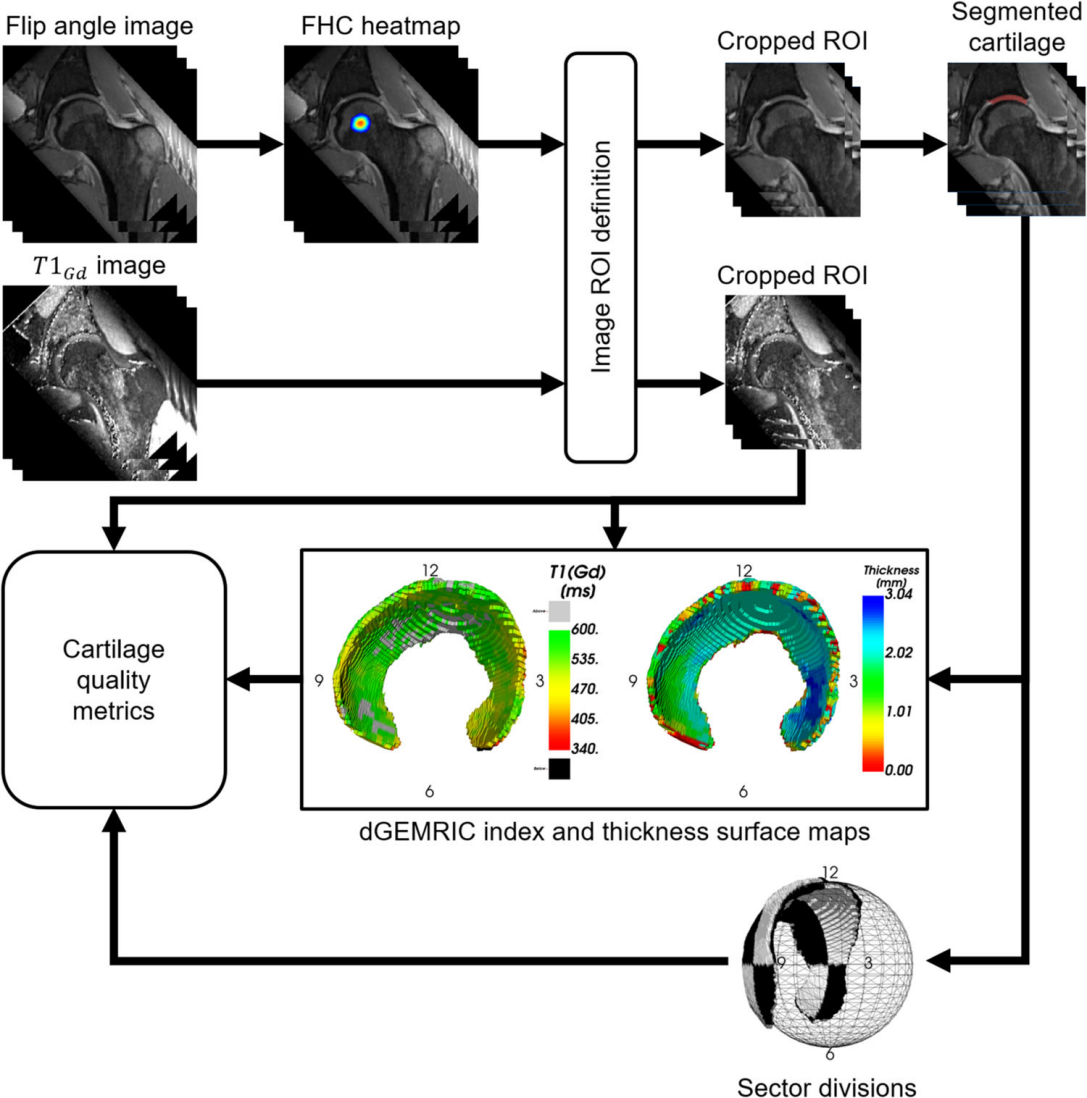
Program pipeline from data loading to cartilage quality metrics computation. FHC: femoral head centre; ROI: region of interest

A custom-made software application for the automated segmentation, visualization and analysis of the hip cartilage was developed and verified (Fig. 1). The C++ application is designed to run locally on personal computers and utilizes the following libraries: libtorch (PyTorch, version 1.8.2, RRID:SCR_018536), Insight Segmentation and Registration Toolkit (ITK, version 5.2.1, RRID:SCR_001149), Visualization Toolkit (VTK, version 9.0.3, RRID:SCR_015013), and Qt (Trolltech, version 5.15.2).

### Patient data

Following approval of all experimental protocols by the Cantonal Ethics Committee of Bern, Switzerland (KEK-Nr. 171/12), algorithm development and evaluation was performed on 25 MRI datasets. The datasets were from 23 patients (10 males and 13 females) with an average age of 31 ± 9 years (range, 20-48 years) with symptomatic hips who underwent diagnostic study comprising a contrast-enhanced MRI at 3T, including a 3D dGEMRIC sequence to assess intra-articular pathology due to structural hip deformity [14]. Patients were consecutively selected in order to have a representative sample with different hip deformities, which lead to 16 hips (64%) with pistol-grip cam deformity, six hips (24%) with acetabular dysplasia, 10 hips (40%) had a pincer deformity (4 hips due to a deep acetabular socket, 6 hips due to acetabular retroversion). Image datasets were acquired with a standard dual-flip angle (DFA) using an isovoxel 3D gradient-echo technique on a 3T scanner (Trio; Siemens, Erlangen, Germany) [27] using a 6-channel flexible body-matrix phased-array coil. The acquired voxel size of $0.83 \times 0.83 \times 0.8$ mm was interpolated to $0.24 \times 0.24 \times 1$ mm producing axial reformatted images. Subsequently, the 25 MRIs were resampled to strict axial planes in LPS (left, posterior, superior) orientation, while preserving their voxel size of $0.24 \times 0.24 \times 1$ mm. No additional prescan for B1 correction was performed [28]. The gradient-echo images obtained at a flip angle of $24\deg$ are used for automatic cartilage modelling, as they yield a better morphological visualization of cartilage than the actual dGEMRIC map. Since flip angle images represent the raw data for calculating dGEMRIC indices, the image sets are aligned.

Additionally, a smaller set of 20 MRIs was used with the more robust 3D magnetization-prepared 2 rapid gradient-echo (MP2RAGE) sequence to assess the generalizability of the algorithm. Moreover, MP2RAGE MRI enables a more accurate T1 mapping of hip cartilage than the 3D DFA technique at 3.0 T [29]. The MP2RAGE dataset has a voxel size of $0.5 \times 0.5 \times 1$ mm and was reformatted similarly to the DFA MRI resulting in voxel size of $0.24 \times 0.24 \times 1$ mm.

### Cartilage localization and segmentation

A two-step automatic segmentation algorithm was developed for 3D modelling of the cartilage. Segmentation is performed on a cropped subvolume of the original image data to increase efficiency and to reduce background complexity. Automatic cropping is performed around the femoral head centre, localized by a deep learning-based landmark detection algorithm. For both femoral head detection and cartilage segmentation, a U-Net architecture [30] based on the implementation of Buda et al. [31] was used with a receptive field width of 140 pixels. It comprises four levels of blocks containing two convolutional layers with Rectified Linear Unit (ReLU) activation and one max pooling layer during encoding and up-convolutional during decoding. The Adam optimizer is with an initial learning rate of 0.001 for the head centre localization networks and 0.01 for the segmentation networks. A learning rate scheduler reduced the learning rate by a factor 10 when detecting a plateau on the training loss. The axial and coronal femoral head detection networks were trained for 150 and 60 epochs, respectively. The axial, coronal, and sagittal segmentation networks were trained for 150, 120, 120 epochs, respectively.

For femoral head localization, the U-Net was trained to predict the distance of each voxel from the femoral head centre. For network training, the 3D location of the femoral head centre was manually picked from each of the 25 image datasets and a 3D heatmap representing the distance of each voxel from the picked centre was generated. To avoid ambiguities in only one plane, and to lower the variability of the prediction, an ensemble of two 2D networks, using the axial and coronal planes, respectively, was used. The predictions were reconstructed to a 3D volume using voxel-wise multiplication, making prediction of a Gaussian-shaped 3D heatmap possible with a 2D U-Net. The location of the maximum value in the 3D heat map was defined as the predicted femoral head centre.

The size of the images was standardized during preprocessing to $768 \times 672 \times 160$ voxels as a trade-off between information loss and unnecessary padding. Because of the reduced accuracy requirement of the femoral head detection, the axial plane images for the axial plane-based model were downsampled to $384 \times 336$ pixels, to increase the training and prediction speed. In addition to the ensemble, data augmentation and early stopping were used to increase the generalization capabilities.

Prior to segmentation, each image slice is cropped to $368\times 368 \times 96$ voxels around the femoral head centre. The area was defined to be sufficiently large to ensure that the cartilage is always contained within the cropped volume.

**Fig. 2:**
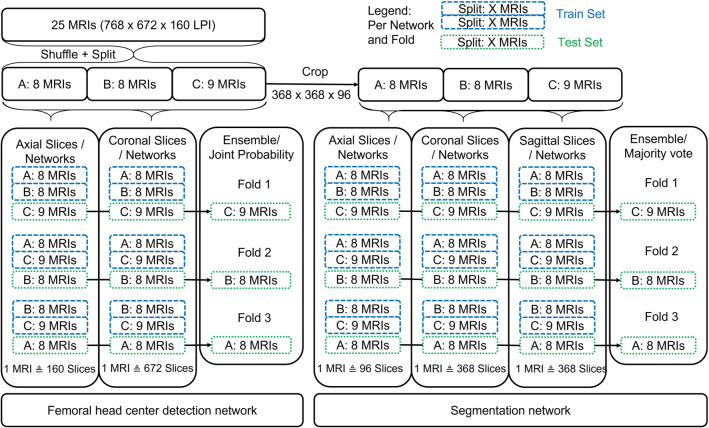
Twenty-five manually segmented axial 3D dual-flip angle MRIs, additionally resliced into coronal and sagittal images, were used for network training and testing. The femoral head detection network is an ensemble of two networks trained with axial and coronal images, respectively. The segmentation network is an ensemble of 3 networks individually trained on axial, coronal, and sagittal images, respectively. For each fold, one-third of the dataset was retained for use as unseen test data. The remaining two-thirds were used for network training

A second network was trained to segment the cartilage from the cropped image datasets. A 2D solution was preferred to a 3D network because it is faster, has a lower memory footprint, and allows a greater number of training samples to be used from a given dataset. For training, clinical experts manually segmented each of the 25 datasets on axial images, while visually confirming accurate segmentation on coronal and sagittal views. Axial, coronal, or sagittal slices were extracted from the volume and used for the network training. To improve segmentation accuracy, three 2D networks were trained with each of the three image planes. Combining the three networks with a voxelwise majority vote, created an ensemble of an axial, sagittal, and coronal plane-based network. No downsampling was used. To reduce the generalization error for this network, data augmentation and early stopping were used. The largest connected object was extracted as the segmentation result. The individual amount of 3D images used for training and testing and the number of 2D slices per MRI are listed in Fig. 2.

### Cartilage sectioning

**Fig. 3:**
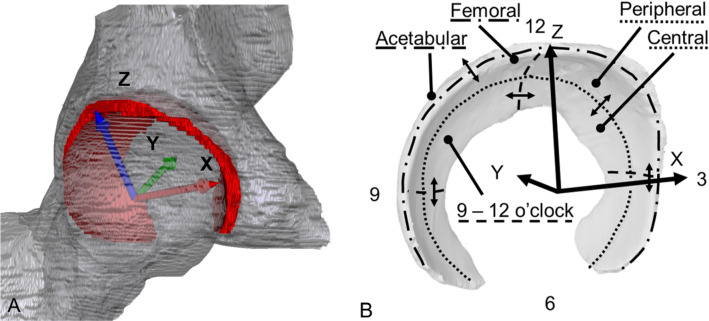
**A** The defined local coordinate system of a right hip depicted with cartilage (red), femoral head, and acetabulum. **B** Based on the local coordinate system and fitted sphere radius, the cartilage is sectioned into a variable number of clock face slices, in this case 4, as well as into central and peripheral sections and into femoral and acetabular layers

The cartilage is sectioned into a variable number of clock face slices as well as into central and peripheral sections and into femoral and acetabular layers (Fig. 3b).

In line with previous definitions, the cartilage sections are defined by a clock face perpendicular to the femoral neck [5, 18, 32]. The acetabular teardrop, which reflects the midpoint of the acetabular notch, defines the 6 o’clock position [14] and the 3 o’clock and the 9 o’clock positions are anterior and posterior in both left and right hips [32].

The centre and radius of the femoral head are automatically determined by fitting a sphere to the 3D segmented cartilage. For this, the least-square sphere estimation algorithm from Yaniv [33] was used, resulting in a sphere fit to the centre of the cartilage layer.

The cartilage follows the rim of the acetabulum. Thus, the clock face is positioned perpendicular to the first mechanical principle axis of the cartilage, *Y*. The axis *Z*, points to the 12 o’clock position, is orthogonal to the axis *Y*, originates at centre of the fitted sphere, and points in the direction of the calculated cartilage centroid. The axis *X* is defined as the cross-product of *Y* and *Z* (Fig. 3).

With the axis *Y* as symmetry axis and axis *Z* as reference axis for the 12 o’clock position, each voxel of the cartilage is assigned to a section. The number of sectors is defined by the user.

As the femoral and acetabular cartilage layers cannot always be visually distinguished in MRI images, especially if traction is not applied to the leg during image acquisition, an automated artificial separation of the acetabular and femoral cartilage was implemented. The radial distance from a specific voxel to the acetabulum or the femoral head surface is measured to categorize if the voxel belongs to the femoral or acetabular layer.

Finally, the cartilage is separated into central and peripheral sections with the central area closer to the acetabular fossa and the peripheral, to the acetabular rim. For an automatic central and peripheral split, a cone is created along the symmetry axis *Y* with tip at the centre of the femoral head.

### Cartilage quality analysis

Automatic algorithms for the calculation of the dGEMRIC index, thickness, surface area, and volume of each 3D cartilage section, were developed.

The dGEMRIC index of a section is defined as the average value of all voxels in the $T1_{GD}$ image within the region defined by the cartilage section. The volume is calculated as the number of voxels in each individual section multiplied by the voxel spacing.

A measure of the load surface, defined as the cartilage surface area excluding unwanted section edges, was developed. To reduce the effects of voxel size, smoothing, and the higher segmentation error typically experienced at the edge of the structure, the fitted sphere, represented by $N = 100'000$ equidistant generated points, was utilized. For each point, an algorithm searches 2 mm radially in and out for the presence of cartilage in the image and assigns the point to the associated section. The individual section surface area, $S_{section}$, is then given by Eq. (1) where $N_{section}$ is the number of points in a given sector associated with sectioned cartilage.

1 $$\begin{aligned} S_{section} = \frac{N_{section}}{N}\cdot 4\pi r^2 \end{aligned}$$

The average thickness of each cartilage section is defined as its volume divided by its surface area. The automatic cartilage modelling and analysis algorithms were integrated into a software application. The software loads the two MRI image datasets in DICOM or NIfTI format as input. The cartilage is localized and segmented without user input. For analysis, the user can define the number of sections on the clockface, enable the femoral and acetabular layers, and the central/peripheral split.

The 3D model of the cartilage is displayed with the dGEMRIC index and thickness rendered on the model surface. For thickness visualization, a 3D distance map is created and voxels are projected to a 2D map (azimuth, inclination) for non-maximum suppression. Similarly, dGEMRIC indices are projected to create a 2D map of radial averages. The acetabular and femoral cartilage layers are averaged individually in two different 2D maps and projected onto the inner and outer surface of the cartilage.

**Table 1:** Comparison between automated cartilage quality metrics calculated from manual and automatic segmented cartilage for four sections

Metric	Overall	12–3	3–6	6–9	9–12
dGEMRIC [ms]	$$-0.8$$ ± 7.9 $$(-4.2 - 2.7)$$ $$p = 0.641$$	$$-2.1$$ ± 10.2 $$(-6.5 - 2.3)$$ *p* = 0.328	$$-3.5$$ ± 26.2 $$(-14.8 - 7.8)$$ $$ p = 0.531$$	4.0 ± 11.4 $$(-0.9 - 9.0)$$ $$p = 0.104$$	$$-0.3 \pm 15.3$$ $$(-2.0 - 5.5)$$ $$ p = 0.244$$
Thickness [mm]	0.0 ± 0.2 $$(-0.1 - 0.1)$$ $$p = 0.461$$	0.0 ± 0.2 $$(-0.1 - 0.1)$$ $$ p = 0.676$$	0.3 ± 0.4 $$(0.1 - 0.5)$$ *p* < 0.001*	0.0 ± 0.3 $$(-0.1 - 0.1)$$ *p* = 0.718	0.0 ± 0.2 $$(-0.1 - 0.1)$$ $$p = 0.622$$
Surface [mm^2^]	48.9 ± 135.6 $$(-7.1 - 104.8)$$ $$p = 0.084$$	13.8 ± 43.1 $$(-4.0 - 31.6)$$ $$ p = 0.123$$	$$-12.6$$ ± 40.7 $$(-29.4 - 4.2)$$ $$ p = 0.134$$	24.0 ± 54.9 $$(1.3 - 46.7)$$ $$ p = 0.039$$*	23.9 ± 53.3 $$(1.9 - 45.9)$$ $$ p = 0.034$$*
Volume [mm^3^]	168.6 ± 624.2 $$(-89 - 426.3)$$ $$p = 0.189$$	27.4 ± 252.7 $$(-76.9 - 131.7)$$ $$ p = 0.592$$	22.4 ± 146.9 $$(-38.2 - 83.0)$$ $$p = 0.453$$	56.9 ± 160.0 $$(-9.2 - 122.9)$$ $$p = 0.088$$	61.9 ± 210.3 $$(-24.9 - 148.7)$$ $$ p = 0.154$$

Mean difference ± SD with 95%, confidence intervals in parentheses, and *p*-values are reported. Number of hips = 25

### Evaluation

The generalization performance of the femoral head centre detection network and the cartilage segmentation network was validated on the 25 datasets in a threefold cross-validation scheme (Fig. 2). The test data were therefore unseen and not used to train the specific network. Femoral head detection accuracy was evaluated as the Euclidean distance between the manually picked ground truth femoral head centre and the predicted centre.

The automatic cartilage segmentation accuracy was evaluated using the Dice similarity coefficient (DSC), the Hausdorff distance (HD), and the average surface distance (ASD) against manual segmentation.

To estimate the segmentation performance on MP2RAGE MRIs the network was trained with all the 25 DFA images and tested on the 20 MP2RAGE images using DCS, HD, ASD against manual segmentation.

To validate the accuracy of the automatic segmentation for use in cartilage analysis, the cartilage quality metrics, calculated on both manual and automatic cartilage segmentation’s for four quadrants, were compared for all 25 DFA image datasets. Normal distribution was evaluated using the Kolmogorov–Smirnov test. Mean differences were calculated and compared with a paired t test or Wilcoxon rank-sum test. RStudio (version 1.4.1717, RRID:SCR_000432) was used for statistical analysis.

## Results

### Cartilage 3D modelling

The trained landmark detection algorithm localized the femoral head centre with a mean and standard deviation accuracy of 4.0 ± 1.8 mm (range, 0.9–7.9 mm). The algorithm thus provides sufficient accuracy for volume cropping (accuracy requirement of 10 mm).

Compared to manual segmentation, the automatic segmentation network predicted the hip cartilage with a Dice similarity coefficient of 88 ± 3% (range, 81–91%), a Hausdorff distance of 4.9 ± 1.5 mm (range, 2.5–9.3 mm), and an average surface distance of 0.2 ± 0.1 mm (range, 0.1–0.4 mm) (*N* = 25).

**Table 2:** Automated cartilage quality metrics calculated from manual segmented cartilage for four sections

Metric	Overall	12–3	3–6	6–9	9–12
dGEMRIC [ms]	569 ± 158	584 ± 167	481 ± 155	523 ± 146	597 ± 163
Thickness [mm]	2.8 ± 0.4	3.3 ± 0.4	3 ± 0.4	2.1 ± 0.4	2.6 ± 0.4
Area [mm^2^]	2028 ± 466	742 ± 163	258 ± 109	329 ± 99	716 ± 147
Volume [mm^3^]	5730 ± 1310	2438 ± 556	777 ± 362	706 ± 268	1809 ± 347

Mean values ± SD are reported. Number of hips = 25

**Fig. 4:**
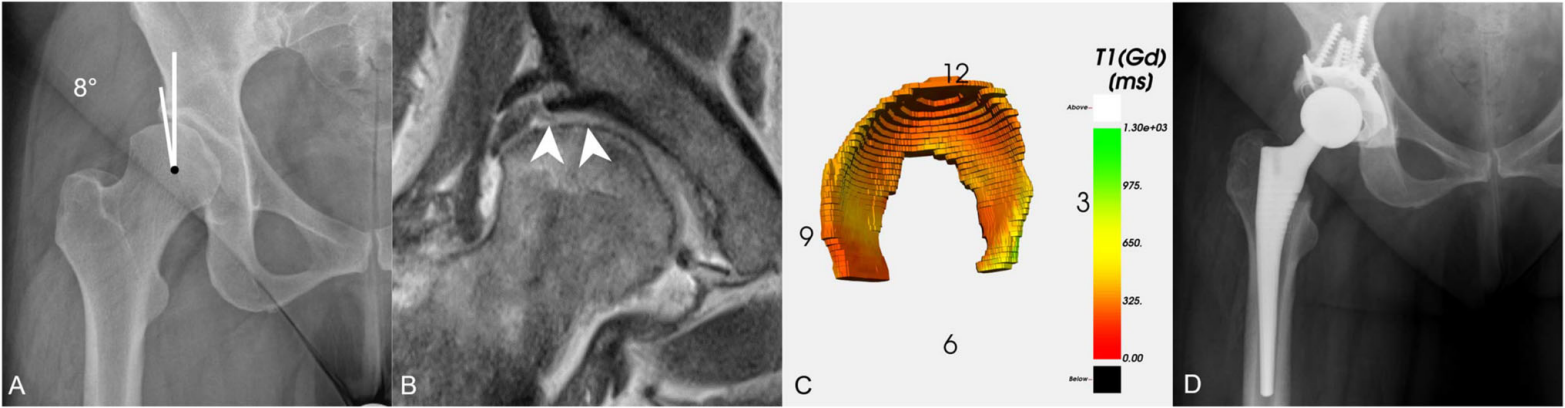
**A** Middle-aged woman with severe hip dysplasia (lateral centre edge angle of 8$^\circ$) and only mild radiographic signs of osteoarthritis. **B** Hip MRI showed focal femoroacetabular cartilage thinning but no obvious signs of general joint degeneration. **C** 3D cartilage model with colour-coded dGEMRIC indices shows generalized decreased dGEMRIC indices (overall mean 358 ms) corresponding to severe biochemical cartilage damage. **D** The patient underwent total hip arthroplasty

**Fig. 5:**
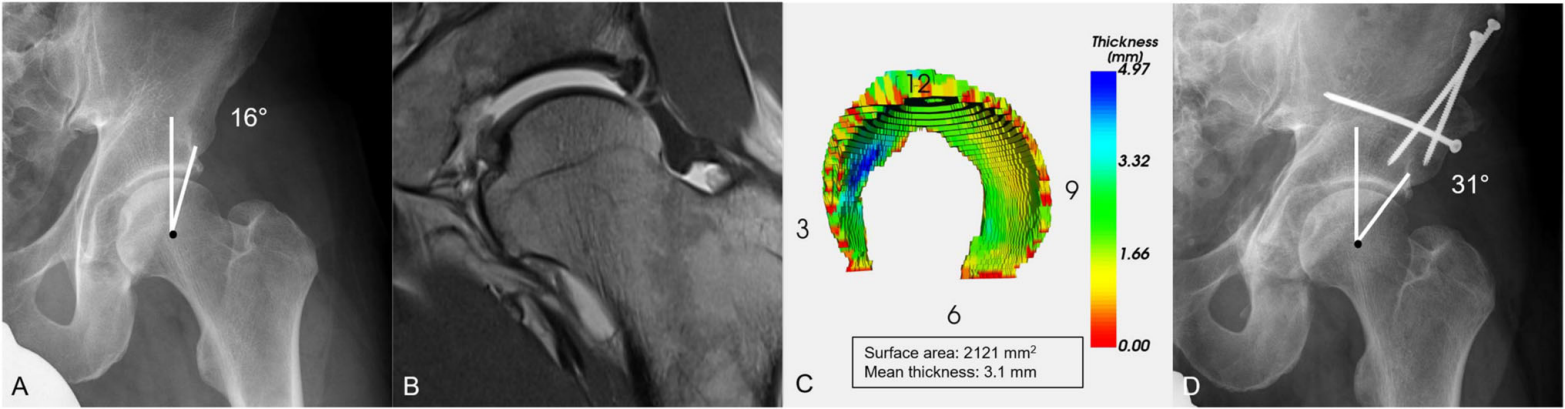
**A** Patient with hip dysplasia (lateral centre edge angle of 16$^\circ$). **B** Hip MRI indicated reduced acetabular coverage. **C** 3D cartilage model with colour-coded means of cartilage thickness shows a small surface area of 2121 mm$^2$ and a high cartilage thickness, typically seen in hip dysplasia. **D** Periacetabular osteotomy was performed to increase acetabular coverage (lateral centre edge angle = 31$^\circ$), i.e. to correct for hip dysplasia

On the additional MP2RAGE dataset, the network performed with a Dice similarity coefficient of 83 ± 4% (range, 70–87%), a Hausdorff distance of 15.5 ± 4.6 mm (range, 9.5–30.6 mm), and an average surface distance of 0.5 ± 0.4 mm (range, 0.2-1.9 mm) against manual segmentation ($N = 20$).

### Cartilage quality analysis

No significant difference in the values of dGEMRIC index and volume, calculated based on the manual and automatic segmentations, was found (*p* >0.05). No overall significant difference in surface area and volume was observed; however, a significant error in thickness of 0.3 ± 0.4 mm was observed in the smallest sector and a significant surface area error of 24.0 ± 54.9 mm$^{2}$ in sector 3–6 and 23.9 ± 53.3 mm$^{2}$ in sector 9–12. The mean errors and standard deviation for each metric per section are summarized in Table 1. The calculated metrics for the 25 hips are given in Table 2. The graphical representations of dGMERIC index and thickness in Fig. 4c and 5c show the overall distribution and critical areas on the cartilage.

### Cartilage quality system

A complete biochemical and morphological analysis of the cartilage is generated by the software in 18 s (15 s for the segmentation pipeline) with a supported graphics processing unit (Octa core Intel i7-9700k, Nvidia RTX2080 Ti) and in 4 min without a GPU (Octa core Intel i7-9700k) and 11 min on a standard laptop (Quad core Intel i7-8550U).

The software system was used to retrospectively evaluate the cartilage of two patients after surgical treatment for hip dysplasia. In one case, dGEMRIC indices, predictive of poor surgical outcome ($<370$ ms) [8], were calculated and displayed by the system (Fig. 4). This information would not have been determined from a radiograph or standard diagnostic MRI. Such an analysis would have further supported treatment by total hip arthroplasty. In case 2, the software calculated a small cartilage surface area and high thickness, typical in hip dysplasia (Fig. 5).

## Discussion

Within this work, we propose a system for efficient automatic analysis of the 3D hip cartilage. The variable sized analysis sectors enable a comprehensive study of the cartilage for identification of better diagnostic parameters, thresholds, and normalization approaches for optimal treatment decision making.

In contrast to other joints [34], the weight bearing cartilage layer in the hip is only 3–4 mm thick and oriented oblique to standard imaging planes increasing the difficulty of segmentation. The implemented pipeline based on the efficient 2D U-net, segments the cartilage 4 times faster with a slightly better accuracy, on an identical dataset, than the network described by Schmaranzer et al. [14] (88% compared to 86% Dice similarity coefficient within 15 seconds compared to approximately 1 minute). The dice similarity coefficient of 83% on the MP2RAGE is lower than on the DFA dataset but shows that the network can generalize over different modalities and can be retrained on new modalities. In the future, we will train the networks on a bigger MP2RAGE dataset to achieve a high segmentation accuracy with accurate T1 mappings. While these results are promising, deep learning-based segmentation methods can experience reduced accuracy on cases not represented in training data. Additional analysis on a larger dataset is required to further assess the generalizability of the algorithm for use in clinical practice. To improve robustness and confidence in the system, methods for case specific, real time, segmentation uncertainty assessment, and guided correction are required. Methods for the identification of sufficient segmentation accuracy, based on the sensitivity of the calculated cartilage quality metrics, are currently being developed.

The data used for training and testing cartilage segmentation were taken from a single centre. Differences in system calibration and protocols across centres can also reduce the segmentation accuracy. Domain adaption techniques are being developed to allow the optimization of the segmentation network for a specific centre without the need for time consuming manual MRI annotations [35].

The automated cartilage quality metrics presented herein, standardizes measures and renders them feasible for use in clinical practice. No significant difference of automatically calculated values of average dGEMRIC index and volume, compared to values presented by Schmaranzer et al. [14], was found. The measured values of thickness differed significantly from previous reported values [14], most likely because Schmaranzer et al. measured cartilage indirectly as the distance between the acetabulum and the femoral head rather than the thickness of the segmented cartilage.

Similar to Schmaranzer et al. [14], significant differences in thickness and the surface area for some sections between manual and automatic segmentation (*p*-value $<0.05$) were observed, demonstrating the sensitivity of these metrics to the segmentation result. For the overall surface, a 95% confidence interval in the range of $-0.7$ to 104.8 mm$^{2}$ was observed. This is smaller than the difference observed in diagnostic groups reported by Steppacher et al. [21], and thus, our approach should enable differentiation of hip deformities according to size of the cartilage surface area. The difference in thickness in one section was significant but within the resolution of one voxel. Further investigation into the sensitivity of cartilage thickness and surface area as predictive treatment outcome measures are required to assess the clinical significance of this uncertainty in measure.

An absolute accuracy evaluation of morphological measures of the cartilage could not be conducted in this study because evaluation was performed on patients. In the future, an analysis on cadaveric specimen would enable verification of the presented automatic quality metric calculations to absolute values.

In contrast to other joints such as the knee, the femoral and acetabular cartilage layers are in direct contact, making identification of the layers difficult. Some researchers have proposed leg traction to achieve a distinct visualization of the cartilage layers within the MRI data [36], while others have proposed the use of deformable shape models [23]. In this work, we have proposed to separate the cartilage along its midline. This approach, being efficient and robust, allows for normalization of the dGEMRIC index and identification in biochemical properties of the cartilage layers.

## Conclusion

A more sensitive and specific diagnosis of cartilage quality through biochemical and morphological assessment in MRI has potential to support treatment decision making for structural hip pathologies. The work presented within represents the first complete system for automatic cartilage modelling and analysis, incorporating both regional analysis and layer differentiation. Ongoing analysis of the predictive power of the calculated measures in a longitudinal study of the outcome of preservation surgery, may lead to the identification of improved diagnostic measures to improve the timing and patient selection for preservation hip surgery, reducing the rate of long-term failure and improving patient outcomes. Allowing a complete assessment in 18 seconds on standard medical imaging data, with minimal user interaction, the application is suitable for routine clinical use, complementing existing radiographic analysis including the accurate MP2RAGE dGEMRIC sequence. This paves the way for the state-of-the-art diagnosis for a variety of hip deformities.

## Data Availability

Code is available on reasonable request.
